# Biomemristic Behavior for Water-Soluble Chitosan Blended with Graphene Quantum Dot Nanocomposite

**DOI:** 10.3390/nano10030559

**Published:** 2020-03-20

**Authors:** Lei Li

**Affiliations:** 1 HLJ Province Key Laboratories of Senior-Education for Electronic Engineering, Heilongjiang University, Harbin 150080, China; lileidtk@hlju.edu.cn; Tel.: +86-136-7462-1831; 2 Research Center for Fiber Optic Sensing Technology National Local Joint Engineering, Heilongjiang University, Harbin 150080, China

**Keywords:** biomemristic behavior, water-soluble CCS:GQDs nanocomposites, CCS-based binary biomemory

## Abstract

Bionanocomposite has promising biomemristic behaviors for data storage inspired by a natural biomaterial matrix. Carboxylated chitosan (CCS), a water-soluble derivative of chitosan avoiding the acidic salt removal, has better biodegradability and bioactivity, and is able to absorb graphene quantum dots (GQDs) employed as charge-trapping centers. In this investigation, biomemristic devices based on water-soluble CCS:GQDs nanocomposites were successfully achieved with the aid of the spin-casting method. The promotion of binary biomemristic behaviors for Ni/CCS:GQDs/indium-tin-oxide (ITO) was evaluated for distinct weight ratios of the chemical components. Fourier transform infrared spectroscopy, Raman spectroscopy (temperature dependence), thermogravimetric analyses and scanning electron microscopy were performed to assess the nature of the CCS:GQDs nanocomposites. The fitting curves on the experimental data further confirmed that the conduction mechanism might be attributed to charge trapping–detrapping in the CCS:GQDs nanocomposite film. Advances in water-soluble CCS-based electronic devices would open new avenues in the biocompatibility and integration of high-performance biointegrated electronics.

## 1. Introduction

Memristic devices, beneficial to high density, large scalability, low power consumption, high endurance and retention performance, have emerged as promising candidates for future high-performance nonvolatile data memory [1,2,3]. They possess a capacitor-like two-terminal metal-insulator-metal (MIM) configuration, where an insulating material is sandwiched between two conductive electrodes. Natural biomaterials offer remarkable building blocks for exploitation in next-generation biosustainable electronics, such as in organic thin-film transistors [4], organic displays and light-emitting devices [5,6], and organic photovoltaics [7]. They provide these devices with environmental benignity, high performance and large-scale fabrication capability at low cost. The nature of biomaterials paves the way for next-generation ultrahigh density and high-speed green data storage devices [8,9,10,11].

As a large constituent of the polysaccharide family, chitosan (CS) bears excellent potential for the development of biointegrated electronic devices, in application to sensor skins, biomedical diagnosis and therapy, and brain-machine interfaces [12,13,14,15,16,17,18]. Its appealing properties consist of biocompatibility, biodegradatiblity, bioresorbability, natural abundance and light weight [19,20]. Unfortunately, some toxic or pungent solvents like trifluoroacetic and acetic acid have been employed for the CS dissolution process. Consequently, acidic salts are generated during the solution-processable method which must be removed in the following experiment. Thus, carboxylated chitosan (CCS) is more environmentally friendly, and has better biocompatibility, with water serving as its solvent. Graphene quantum dots (GQDs) are graphene nanosheets of usually less than 10 nm in size. Advances in GQDs make them suitable for optoelectronic applications, due to their small size, attractive optical properties, biocompatibility and low preparation cost. GQDs can hold charge storage in the trapping level under the exciton confinement and quantum size effects [21]. The carriers cannot be effectively transported to electrodes as a result of their poor coupling with each other. A method blending inorganic nanoparticles into natural biomaterials has opened up a new way to create biomemristic materials.

Great efforts to develop biocompatible or biodegradable devices have been made, adopting both organic materials and inorganic materials [13,22]. However, the balance between device performance and biocompatibility or biodegradability has been insufficiently considered. The biodegradability and biocompatibility requirements predominantly impose restrictions on the suitability of conductive materials for complementary metal-oxide−semiconductors (CMOS), such as Pt, Ag, Al, Si, etc. [23]. Therefore, it is crucial to rebuild bioelectronic systems to achieve a trade-off between device performance and biocompatibility. Nevertheless, biomemory devices based on biocompatible materials like protein, cellulose and DNA exhibit binary resistive-switching behavior, with biologically incompatible electrodes like Al, Pt, Au and Ag [24,25,26]. This may hinder the implementation of a practical biomemory system. This paper aims at providing a novel biomemristic device using Ni/CCS:GQDs/indium-tin-oxide (ITO), in which CCS:GQDs nanocomposites serve as passive components; ITO and Ni are used as top and bottom electrodes, respectively. CCS:GQDs nanocomposites are nontoxic, sustainable, and environment-friendly, and, furthermore, ITO and Ni are nontoxic and non-polluting for the environment and can be recycled and reused. The focus is on the charge trapping–detrapping mechanism concerning CCS:GQDs nanocomposites as well, which renders them promising candidates for enabling the biocompatibility and integration of high-performance biomemristic devices.

## 2. Materials and Methods

CCS (*M*_n_ = 48 kg/mol) was purchased from Aladdin (Tianjin, China). The aqueous solution of GQDs (1 mg·mL^−1^) was obtained from Tanfeng Tech. Inc (Suzhou, China). For fabrication of biomemristic devices, homogeneous CCS:GQDs nanocomposite solutions (10 mg·mL^−1^) with GQD contents of 1 wt%, 3 wt%, and 5 wt%, respectively, were prepared in deionized water by means of ultrasonication. The glass substrates were precleaned by a sonication-aided washing process in which the washing solvents covered acetone, absolute alcohol, and deionized water. They were then dried in an oven (Zhonghuan Furnace, Tianjin, China) at 40 °C. Afterwards, a spin-casting process was employed at a speed of 2000 rpm/40 s, in which the CCS:GQDs nanocomposite solution was spun onto the precleaned glass slides coated with an ITO layer (the square resistance *R*_s_ ≤ 6 Ω/sq). Then, the CCS:GQDs nanocomposite films were dried on a hot plate at 60 °C for 1 h. By thermal evaporation, Ni electrodes were deposited onto biofilms (coated on the substrates) at a pressure below 10^−5^ Torr. The top metal electrode was determined to have a thickness of 200 nm, and a size of up to 1.0 × 1.0 mm^2^.

The thermal, structural, and morphological property characterizations and comparisons of CCS:GQDs nanocomposites were carried out by thermogravimetric analyses (TGA), fourier transform infrared spectroscopy (FTIR), Raman spectroscopy and scanning electron microscopy (SEM). Thermal properties of CCS:GQDs nanocomposites were taken into account, which were characterized by TGA (TA Instruments, New Castle, DE, USA) under N_2_ at a heating rate of 10 °C/min. A Foss DS 2500 Infrared Spectrometer (Hillerød, Denmark), using KBr pellets, was employed to test FTIR spectra swept from 400 cm^−1^ to 4000 cm^−1^, to elucidate functional groups of CCS:GQDs nanocomposites. Raman spectroscopy (Horiba Jobin Yvon, Villeneuve-d’Ascq, France), scanned from 100 cm^−1^ to 3200 cm^−1^, was utilized to detect the structure of CCS:GQDs nanocomposites. The morphological and cross-sectional profiles of the nanocomposite films with GQDs embedded into CCS were additionally characterized by an Apreo Scanning Electron Microscope (Themoscientific, Waltham, MA, America). This compared the morphologies of the CCS:GQDs nanocomposite films with biomemristic behaviors. Electrical measurements without any device encapsulation were fulfilled by a Keithley 4200 semiconductor parameter analyzer (Solon, OH, USA).

## 3. Results

**Figure 1:**
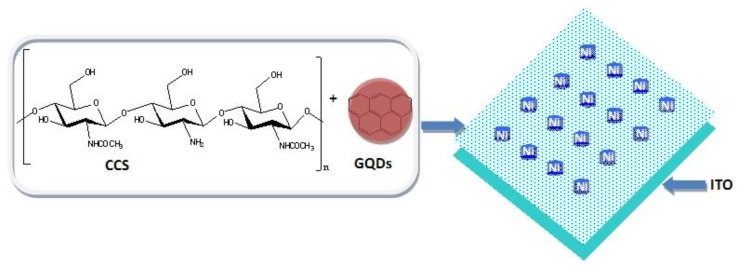
Schematic structure of carboxylated chitosan (CCS) and graphene quantum dots (GQDs), and configuration of the sandwiched biomemristic device Ni/CCS:GQDs/ITO.

**Figure 2:**
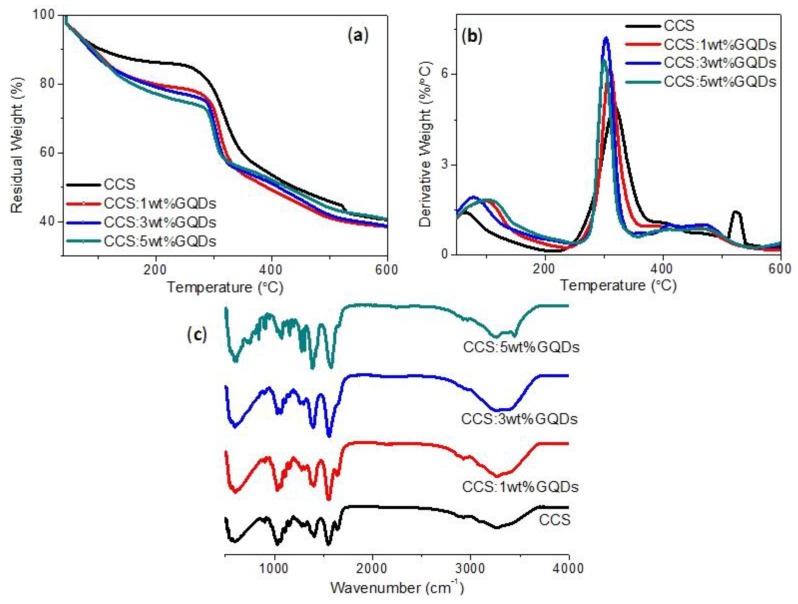
(**a**) TGA, (**b**) DTG and (**c**) FTIR spectra of pure CCS, and CCS:GQDs nanocomposites.

The sandwiched MIM configuration for the biomemristic device Ni/CCS:GQDs/ITO was presented in Figure 1, together with the schematic structure of CCS and GQD. To study the thermal stability of CCS and CCS:GQDs nanocomposites, TGA–DTG was investigated under an N_2_ atmosphere. TGA–DTG curves are shown in Figure 2a,b, in which different mass loss steps are displayed. CCS and its nanocomposites predominately showed three mass loss steps [27,28]. CCS:GQDs nanocomposites exhibited a dehydration mass loss step ascribed to water loss from the surface of CCS. After dehydration, the decomposition of CCS and its nanocomposites with GQD contents of 1 wt%, 3 wt% and 5 wt% occurred in two steps, starting at 287.0 °C, 295.9 °C, 291.8 °C, and 290.8 °C, respectively. It was witnessed that CCS:GQDs nanocomposites were more stable than CCS because of higher initial temperatures of thermal degradation [29]. After dehydration, the other mass loss steps were attributed to thermal decomposition of the polymer chain [30]. Table 1 provides a description of events, temperature intervals and quantitative data for CCS and CCS:GQDs nanocomposites. The proportionality between the second and first steps after water loss was also calculated (Table 1) [27,28]. Compared with CCS, it was observed that the ratio between the mass losses in the second and first step of decomposition was augmented from 0.6 to 1.0 for CCS:GQDs nanocomposites with content of GQDs increasing from 1 wt% to 5 wt%. Therefore, the results showed that CCS:GQDs nanocomposites had similar decomposition process but were more stable in contrast with CCS.

CCS has abundant amine groups in its structure, which can interact with hydroxyl groups of GQDs by hydrogen bonding. For the presence of functional groups for CCS and CCS:GQDs nanocomposites, FTIR spectra were tested for the samples before and after blending with GQDs, as shown in Figure 2c. For FTIR spectra of pure CCS, it illustrates the OH stretching band at 3264 cm^−1^, the amide I band at 1641 cm^−1^, the amide II band at 1550 cm^−1^, the bridge oxygen stretching band at 1152 cm^−1^, and the C–O stretching bands at 1061 cm^−1^ and 1027 cm^−1^ [31]. The 1560/1070 peak ratio was used to determine the percent deacetylation of CCS-based samples [32]. On the basis of the FTIR spectra for CCS and its nanocomposites with GQD contents of 1 wt%, 3 wt% and 5 wt%, the samples were 94%, 74%, 57%, 49% deacetylated, respectively, as reckoned in Table 2. As a result of the interaction between CCS and GQDs, significant changes were observed in the FTIR spectra. FTIR spectra of CCS:GQDs nanocomposites presented a distinct change in the carbonyl-amide region. The primary amine peak decreased while a new peak for C=N imine appeared [33]. Moreover, the C=N peak appeared as a strong split peak at 1650 cm^−1^. The addition of GQDs incrementally, from 1 wt% to 5 wt%, to CCS resulted in conformational changes, such as: the peaks of CCS at 3264 cm^−1^ shifting to 3270 cm^−1^, 3262 cm^−1^, and 3259 cm^−1^, respectively; the peak at 1550 cm^−1^ shifting to 1550 cm^−1^, 1557 cm^−1^, and 1578 cm^−1^, respectively; the peak at 1401 cm^−1^ shifting to 1400 cm^−1^, 1397 cm^−1^ and 1386 cm^−1^, respectively; the peak at 1027 cm^−1^ shifting to 1028 cm^−1^, 1028 cm^−1^, and 1030 cm^−1^, respectively. This is possibly due to the interaction between CCS and GQDs. FTIR spectra of CCS:GQDs nanocomposites with different amounts of GQDs were similar to that of CCS. However, the absorption peak at 3264 cm^−1^ slightly widened and moved by blue shift, which might be attributed to the overlapping of O–H and NH^−^ stretching vibrations [34]. Stemming from the interaction between GQDs and CCS, the change altered the chemical environment of hydrogen bonds between CCS molecules, GQDs and amino groups of CCS [35].

**Figure 3:**
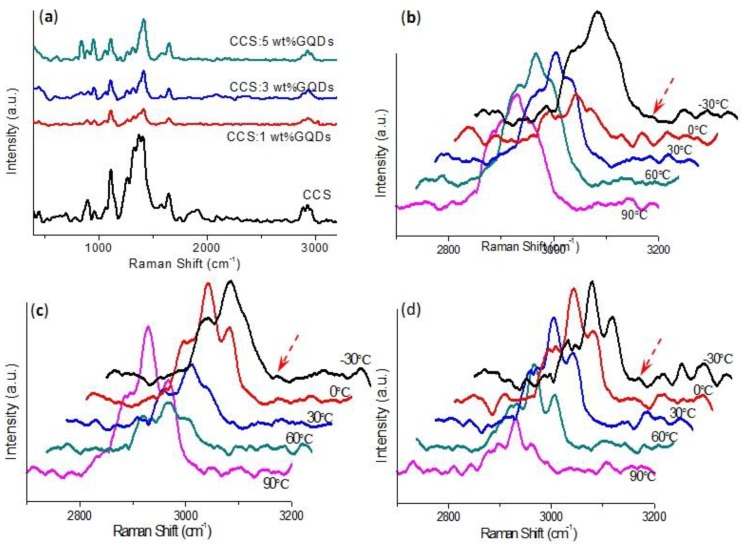
(**a**) Raman spectra of CCS and its nanocomposites with distinct chemical ratios of GQDs at 25 °C. Temperature dependence of Raman spectra for CCS:GQDs nanocomposites with GQD contents of (**b**) 1 wt%, (**c**) 3 wt% and (**d**) 5 wt%.

**Figure 4:**
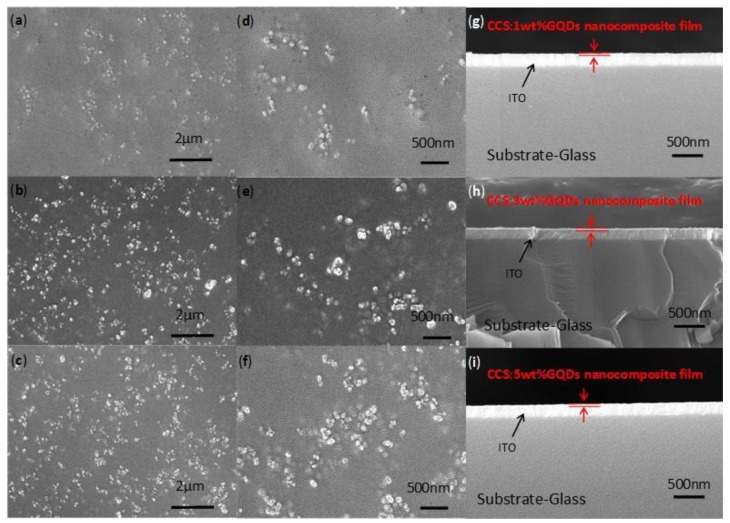
Morphological characterization of CCS: 1 wt% GQDs, CCS: 3 wt% GQDs and CCS: 5 wt% GQDs nanocomposites based on SEM with magnification of (**a**–**c**) 40,000 and (**d**–**f**) 100,000. (**g**–**i**): the cross-section of CCS:GQDs nanocomposite films with GQD contents of 1 wt%, 3 wt%, 5 wt%, respectively.

To make a further investigation of the composition of CCS:GQDs nanocomposites, Raman spectroscopy was carried out by the 532 nm line of an argon laser, tested in the spectral range from 100 cm^−1^ to 3200 cm^−1^. In a sample volume of 200 μm^3^, using a 50 × objective, the Raman signal was utilized to analyze the temperature dependence of Raman spectra. As indicated in Figure 3, this was to unveil whether there existed hydrogen bonding in CCS:GQDs nanocomposites. As shown in Figure 3a, Raman spectra of CCS and CCS:GQDs nanocomposites at room temperature were represented by three wide regions that ranged between 800 cm^−1^ and 970 cm^−1^, between 1000 cm^−1^ and 1200 cm^−1^, and between 1240 cm^−1^ and 1500 cm^−1^ [36]. In the first region, two peaks for CCS that appeared at 907 cm^−1^ and 966 cm^−1^ were contributions of NH_2_ wagging facilitating the peaks for CCS:GQDs nanocomposites at 905 cm^−1^ and 963 cm^−1^, 905 cm^−1^ and 963 cm^−1^, and 900 cm^−1^ and 957 cm^−1^, respectively [37,38]. The second region revealed the presence of three peaks for CCS at 1067 cm^−1^, 1115 cm^−1^ and 1152 cm^−1^, which corresponded to C–C stretching vibrations of all-trans segments, C–C stretching vibrations of the gauche conformer, and C–C stretching vibrations of the trans conformer, respectively [37]. For CCS:GQDs nanocompostes, the corresponding peaks were at: 1067 cm^−1^, 1112 cm^−1^ and 1152 cm^−1^ for CCS 1 wt% GQDs; 1061 cm^−1^, 1112 cm^−1^ and 1149 cm^−1^ for CCS 3 wt% GQDs; 1066 cm^−1^, 1113 cm^−1^ and 1151 cm^−1^ for CCS 5 wt% GQDs. Three peaks for CCS observed at 1375 cm^−1^, 1416 cm^−1^ and 1460 cm^−1^ within the third region were ascribed to the CH bending, wagging and twisting of CH_2_ [36,39]. For Raman spectra of CCS:GQDs nanocomposites, Figure 3b–d clearly reveal a very subtle merging intensity around 3040 cm^−1^ in the freeze-dried product (−30 °C). This additional band in the freeze-dried sample was detected in the special region where N–H stretching bands were Raman active between C–H and O–H stretching bands. This might be caused by intermolecular motions involved in intermolecular associations via hydrogen bonding [40]. Through hydrogen bonding between CCS and GQDs, the successful nanocomposite formation could be verified [24]. To further explore the morphological and cross-sectional profiles of CCS:GQDs nanocomposite films, the surface microstructure was observed by SEM (Figure 4a–f). Discontinuous bulges arose on the surface of CCS:GQDs nanocomposite films. The addition of GQDs modified the microstructure of the CCS-based films by increasing the surface bulges (Figure 4b,c). Moreover, compact structures were observed in the cross-sections of CCS:GQDs nanocomposite films, as indicated in Figure 4g–i. These bulges might be attributed to the hydrogen bonding interaction between CCS and GQDs, because CCS might act as a binder to bind GQDs, which may then form aggregates to make a difference to the morphology [24].

**Figure 5:**
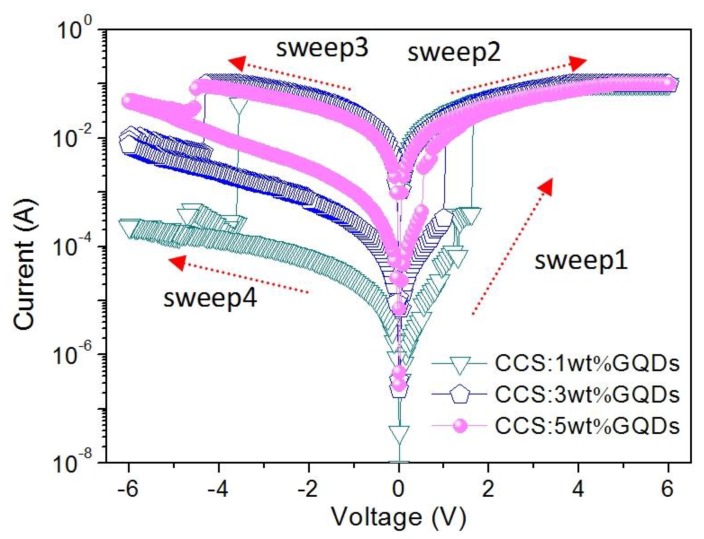
*I*–*V* characteristics of the bistable biomemristic behaviors of CCS blended with distinct GQD contents of 1 wt%, 3 wt%, and 5 wt%, respectively.

In this work, biomemristic behaviors of Ni/CCS:GQDs/ITO can be observed in Figure 5, in which the compliance current of 0.1 A was set up to avoid permanent electrical breakdown. The arrows in the diagram demonstrate the cyclic scanning directions of the applied biases in turn. For Ni/CCS:1 wt%GQDs/ITO, when a voltage was initially applied from 0 V to 1.6 V (sweep 1), a relatively low current was observed. The corresponding resistance level could be defined as a high resistance state (HRS) or OFF-state. When the sweeping bias exceeded 1.6 V (*V*_SET_), a current increase suddenly arose, indicating that the device switched from an HRS to a low resistance state (LRS) or ON-state. This switching corresponded to a “SET” process. Moreover, the device was kept in an LRS even if the applied bias continuously increased up to 6 V. Subsequently, a positive voltage was swept from 0 V to 6 V (sweep 2) once again, indicating that the device still remained stable in an LRS. When the applied bias was continuously scanned from 0 V to the negative voltage *V*_RESET_ = −3.6 V (sweep 3), the device switched back to HRS. The relative process can be denoted as “RESET”. Then the negative bias was swept from 0 V to −6 V again, when the device was maintained in an LRS (sweep4). An important parameter was the HRS/LRS resistance ratio for binary biomemory applications based on the bistable resistive switching effect. A high HRS/LRS resistance ratio (*R*_HRS_/*R*_LRS_ > 10^3^) was obtained for Ni/CCS:1 wt% GQDs/ITO, which could effectively avoid the error detection in the biomemristic states during the SET and RESET processes. 

**Figure 6:**
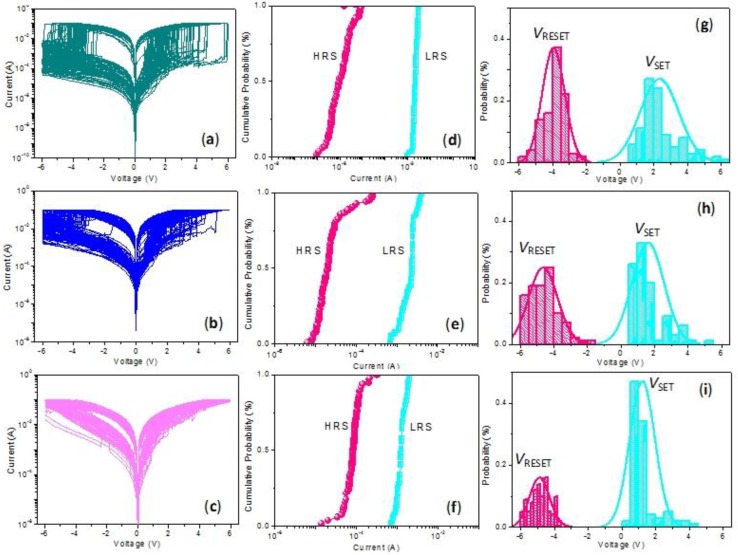
Cycle-to-cycle performance of (**a**) Ni/CCS:1 wt% GQDs/ITO, (**b**) Ni/CCS:3 wt% GQDs/ITO and (**c**) Ni/CCS:5 wt% GQDs/ITO during 100 continuous cycles for binary data storage applications. In detail, (**d**–**f**) show cumulative analyses for the current distribution in the low and high-resistance states (LRS and HRS) during cycle-to-cycle operation. (**g**–**i**) are SET and RESET voltage (*V*_SET_ and *V*_RESET_) distributions for 100 cycles, presented as histograms.

**Figure 7:**
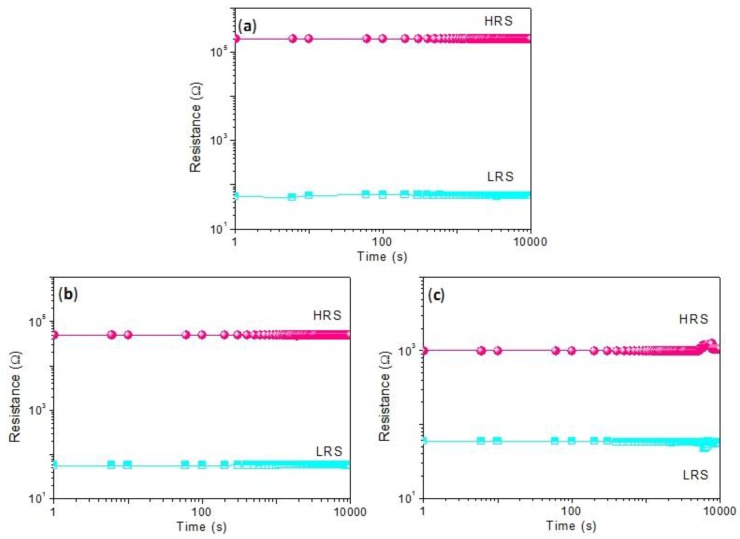
Retention test at a constant voltage of −0.1 V for (**a**) Ni/CCS:1 wt% GQDs/ITO, (**b**) Ni/CCS:3 wt% GQDs/ITO and (**c**) Ni/CCS:5 wt% GQDs/ITO.

Figure 6a exhibits *I*–*V* characteristics over 100 consecutive resistive switching cycles, which proved that the biomemristic behaviors of the device were bistable without obvious attenuation. In addition, the binary biomemristic performance of Ni/CCS:3 wt% GQDs/ITO was tested, whose *V*_SET_ and *V*_RESET_ values were separately 1 V and −4.3 V. Its resistance ratio, *R*_HRS_/*R*_LRS_, could reach ~10^2^. The device was subject to 100 consecutive resistive switching cycles, with *I*–*V* characteristics as shown in Figure 6b. In particular, the obtained *I*–*V* curves for Ni/CCS:5 wt% GQDs/ITO present bistable biomemristic behaviors that display *V*_SET_ = 0.5 V and *V*_RESET_ = −4.45 V, with *R*_HRS_/*R*_LRS_ approaching 30. Its 100 consecutive *I*–*V* characteristics for binary biomemory are indicated in Figure 6c. Furthermore, the cycle-to-cycle performance of CCS:GQDs nanocomposite films was elaborately studied. Cumulative plots of the current in HRS and LRS (*I*_HRS_ and *I*_LRS_) for the cycle-to-cycle operation at *V* = 0.1 V are exhibited in Figure 6d–f. The mean values and standard deviations of *I*_HRS_ and *I*_LRS_ are presented in Table 3. Figure 6g–i represent the distributions of *V*_SET_ and *V*_RESET_ during the cycle-to-cycle operation. The retention behaviors shown in Figure 7 demonstrate the stability of the biomemristic devices in an HRS and an LRS for 10^4^ s. During the retention time, the devices were read at a constant bias of −0.1 V, kept without any substantial electrical degradation. The above data confirm the non-volatile binary biomemristic behaviors of Ni/CCS:GQDs/ITO devices. These results show they meet the requirements of green data storage. 

**Figure 8:**
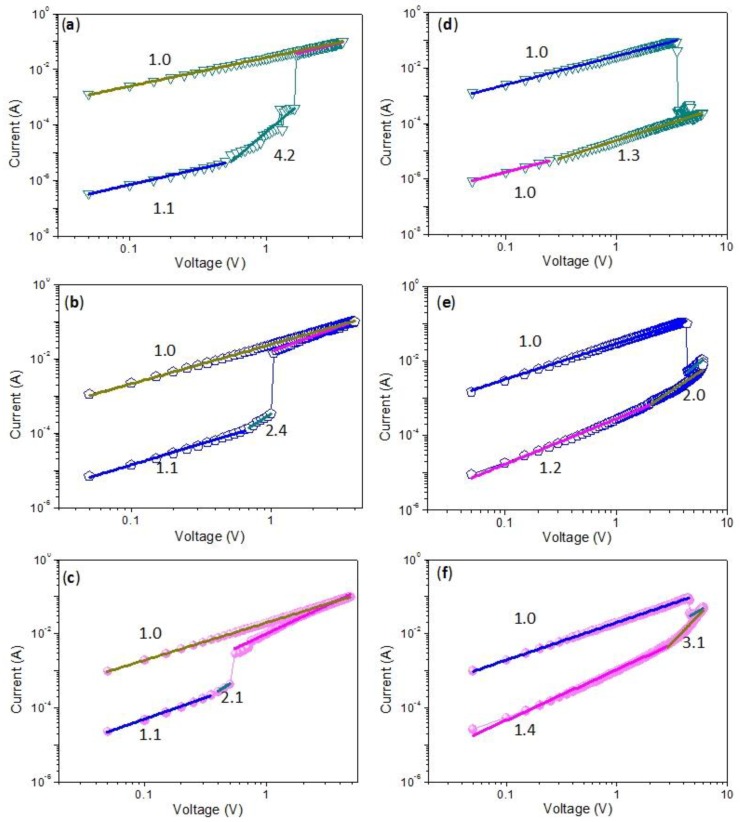
Fitting curves for the binary biomemristic behaviors with different content of GQDs 1 wt%, 3 wt% and 5 wt%, respectively, during the (**a**–**c**) SET and (**d**–**f**) RESET process.

In order to understand the conduction mechanisms of the biomemristic devices, *I*–*V* curves (Figure 5) were plotted in a log-log scale during the SET and RESET process and made by a linear fitting, shown in Figure 8. The slope was ~1 when the device was maintained in the LRS, exhibiting that the device displayed Ohmic conductance. For the HRS, the *I*–*V* relationship became nonlinear as space-charge limited conductance (SCLC) dominated. On the log–log scale, the curve at the low voltage region of the HRS showed a linear relationship, signifying Ohmic conduction, whereas the slope of the *I*–*V* curve increased to 2 or exceeded 2 at the high voltage region, which revealed trap-free or trap-limited SCLC [24,41]. Because CCS behaved like an insulator [42,43,44,45], the fitting curves on experimental data further confirmed that the conduction mechanisms might be attributed to charge trapping–detrapping in the CCS:GQDs nanocomposite film. At a lower voltage, charge transport was limited on account of the insulating barrier provided by the CCS matrix, and GQDs captured the injected charge from the electrode. The injected carriers exponentially increased when the bias exceeded the switching voltage, giving rise to an abrupt growth of the current and transition of the device from the HRS to the LRS. Consequently, almost all the traps were occupied in the LRS, with Ohmic behavior. Moreover, the trapped charges were maintained in the GQDs, even when the devices were powered off. By scanning a reverse voltage, the trapped charges could be detrapped, and the device returned back to the HRS. Therefore, the SET and RESET processes of data storage were performed.

## 4. Conclusions

In summary, this work has demonstrated that water-soluble CCS:GQDs nanocomposite films possess binary biomemristic characteristics with *R*_HRS_/*R*_LRS_ current ratios (memory window) greater than 10^3^. The results fully indicate that the biomemristic devices made from natural bionanocomposites have remarkable potential for green data storage. CCS is nontoxic, sustainable, and environmentally friendly, and ITO and Ni are nontoxic and non-polluting for the environment and can be recycled and reused. This opens up a new way for the next generation of bioelectronic devices, with applications for wearable equipment, medical facilities and implanted devices. 

## Figures and Tables

**Table 1:** Data obtained from TGA–DTG curves of CCS and its nanocomposites.

	N_2_ Atmosphere	TGA–DTG		
		ΔT ^a,b^ (°C)	Mass Loss (%)	Ratio
CCS	Dehydration	45.3–287.0	14.3	
	1st step	287.0–372.3	28.7	0.6 ^b^
	2nd step	287.0–600	16.0	
CCS:1 wt% GQDs	Dehydration	45.3–295.9	22.0	
	1st step	295.9–351.6	24.0	0.6 ^b^
	2nd step	351.6–600	15.2	
CCS:3 wt% GQDs	Dehydration	45.3–291.8	24.2	
	1st step	291.8–338.6	19.9	0.8 ^b^
	2nd step	338.6–600	16.8	
CCS:5 wt% GQDs	Dehydration	45.3–290.8	26.1	
	1st step	290.8–332.5	16.6	1.0 ^b^
	2nd step	332.5–600	16.2	

^a^ Temperature range; ^b^ ratio between mass losses in the second and first step after water loss.

**Table 2:** Evaluation of the percent deacetylation based on FTIR spectra of pure CCS and CCS:GQDs nanocomposites.

	N–H (amine II)		C–O–C	
	ν_N–H (amine II)_ (cm^−1^)	Intensity	ν_C–O–C_ (cm^−1^)	Intensity
CCS	1550	61.3	1062	65.4
CCS:1 wt% GQDs	1550	40.0	1064	53.9
CCS:3 wt% GQDs	1557	35.1	1065	61.3
CCS:5 wt% GQDs	1576	33.7	1065	69.2

**Table 3:** Data profiles concerning the mean and standard deviations (*I*_mean_ and *I*_std_).

	HRS		LRS	
	*I*_mean_ (A)	*I*_std_ (A)	*I*_mean_ (A)	*I*_std_ (A)
CCS:1 wt% GQDs	3.2 × 10^−6^	1.5 × 10^−5^	0.0023	4.7 × 10^−4^
CCS:3 wt% GQDs	8.8 × 10^−5^	4.8 × 10^−5^	0.0022	9.6 × 10^−4^
CCS:5 wt% GQDs	1.3 × 10^−4^	4.9 × 10^−4^	0.0022	9.1 × 10^−4^
